# Telomere Shortening Impairs Regeneration of the Olfactory Epithelium in Response to Injury but Not Under Homeostatic Conditions

**DOI:** 10.1371/journal.pone.0027801

**Published:** 2011-11-16

**Authors:** Masami Watabe-Rudolph, Yvonne Begus-Nahrmann, André Lechel, Harshvardhan Rolyan, Marc-Oliver Scheithauer, Gerhard Rettinger, Dietmar Rudolf Thal, Karl Lenhard Rudolph

**Affiliations:** 1 Department of Otorhinolaryngology, University of Ulm, Ulm, Germany; 2 Max-Planck-Research Department of Stem Cell Aging and Institute of Molecular Medicine, University of Ulm, Ulm, Germany; 3 Department of Pathology, University of Ulm, Ulm, Germany; University of Massachusetts Medical School, United States of America

## Abstract

Atrophy of the olfactory epithelium (OE) associated with impaired olfaction and dry nose represents one of the most common phenotypes of human aging. Impairment in regeneration of a functional olfactory epithelium can also occur in response to injury due to infection or nasal surgery. These complications occur more frequently in aged patients. Although age is the most unifying risk factor for atrophic changes and functional decline of the olfactory epithelium, little is known about molecular mechanisms that could influence maintenance and repair of the olfactory epithelium. Here, we analyzed the influence of telomere shortening (a basic mechanism of cellular aging) on homeostasis and regenerative reserve in response to chemical induced injury of the OE in late generation telomere knockout mice (G3 *mTerc^−/−^*) with short telomeres compared to wild type mice (*mTerc^+/+^*) with long telomeres. The study revealed no significant influence of telomere shortening on homeostatic maintenance of the OE during mouse aging. In contrast, the regenerative response to chemical induced injury of the OE was significantly impaired in G3 *mTerc^−/−^* mice compared to *mTerc^+/+^* mice. Seven days after chemical induced damage, G3 *mTerc^−/−^* mice exhibited significantly enlarged areas of persisting atrophy compared to *mTerc^+/+^* mice (p = 0.031). Telomere dysfunction was associated with impairments in cell proliferation in the regenerating epithelium. Deletion of the cell cycle inhibitor, Cdkn1a (p21) rescued defects in OE regeneration in telomere dysfunctional mice. Together, these data indicate that telomere shortening impairs the regenerative capacity of the OE by impairing cell cycle progression in a p21-dependent manner. These findings could be relevant for the impairment in OE function in elderly people.

## Introduction

The olfactory epithelium (OE) represents a neuroepithelium with low rates of cell turnover but it can regenerate throughout the life span of vertebrates in response to injury or inflammatory damage [1], [2]. The OE consists of three major cell types: olfactory receptor neurons, supporting cells and basal cells [3], [4]. The basal cell layer of the olfactory epithelium contains neuronal progenitor cells generating new receptor neurons throughout life [5], [6].

Dysfunction of the OE (hyposmia, dry nose) is a very frequent clinical symptom in the elderly occurring in >75% of 80 year old people [7]. Several clinical conditions can precipitate OE dysfunction including nasal infections and surgery. Morphologically, OE dysfunction has been associated with reduced thickness of the epithelium and impaired mucosa secretion [8] indicating that regenerative dysfunction and atrophic changes of the OE could contribute to the age associated development of hyposmia. In addition, olfactory dysfunction associates with some neuronal disease including Alzheimer's Disease and Parkinson's Disease [9], [10].

The association between aging and the evolution of OE dysfunction indicates that molecular mechanisms of aging may also impair the homeostasis and/or the regenerative capacity of the OE. It has been postulated that hormonal changes may be involved in the development of OE atrophy [11], [12]. Molecular alterations that contribute to the decline in OE homeostasis and regeneration have yet to be delineated.

Telomere shortening represents one molecular mechanism, which can limit cell proliferation and the regenerative capacity of tissues. Telomeres form the end structures of human chromosomes [13]. They consist of simple tandem DNA repeats and telomere binding proteins [14]. The main function of telomeres is to cap chromosomal ends to prevent chromosomal stability. Telomeres shorten with each round of cell division due to the ‘end-replication problem’ of DNA polymerase and due to processing of telomeres during S-phase [15]. When telomeres reach a critically short length they lose capping function and 3 to 4 dysfunctional telomeres per cell are sufficient to induce the DNA damage response leading to a permanent cell cycle arrest (replicative senescence) or apoptosis [16].

Cell culture experiments have shown that telomere shortening limits the proliferative capacity of primary human cells to a finite number of cell divisions [17]. Telomere shortening has also been shown to impair the proliferative capacity of neuronal stem cells [18]. There is growing evidence that telomeres shorten in various tissues during human aging [19]. Moreover, telomere shortening is accelerated by chronic diseases that increase the rate of cell turnover, e.g. chronic liver disease or chronic HIV infection [20], [21]. Telomerase can synthesize telomeres *de novo* [22]. However, in humans, the expression of the catalytic subunit of telomerase (TERT) is postnatally suppressed in most somatic tissues and this suppression limits telomere maintenance and the proliferative capacity of most somatic cells [23]. During aging, telomeres shorten also in human stem cells indicating that low levels of telomerase are not sufficient to maintain stable telomeres in stem cells during aging [24]. Recent studies have provided evidence that telomerase mutations are the cause of some rare diseases in humans leading to accelerated telomere shortening, organ failure (bone marrow failure, lung fibrosis), and premature death of the patients [25], [26]. Together, these data indicate that human telomeres are limited and can represent the cause of impaired organ maintenance during human aging and disease.

Studies on telomerase knockout mice (lacking the RNA component of telomerase: TERC) have revealed first experimental evidence that telomere shortening limits organ homeostasis and regeneration by induction of cell cycle arrest or apoptosis [27]–[29]. The possible contribution of telomere shortening to maintenance and regeneration of the OE has not been investigated.

Here we analyzed the consequences of telomere shortening in G3 *mTerc^−/−^* mice compared to *mTerc^+/+^* mice with long telomeres on maintenance and regeneration of the OE in response to chemical induced tissue damage. The study shows that telomere shortening leads to regional defects in OE regeneration in response to damage coupled with impaired cell proliferation in the affected areas.

## Results

### Telomere shortening does not impair homeostasis of the olfactory epithelium in aging mice

**Figure 1:**
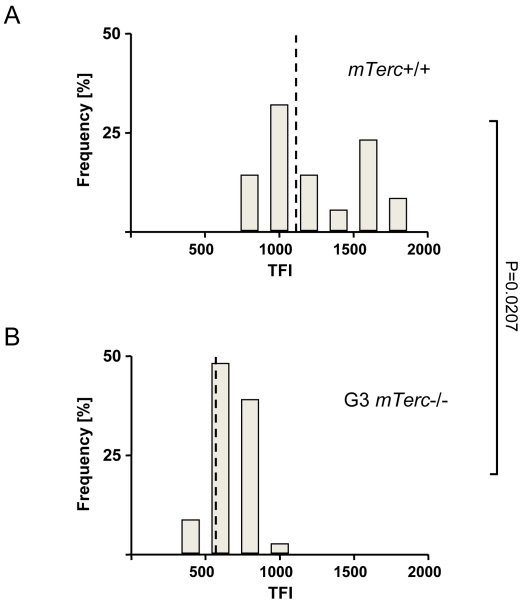
Shortened telomeres in the olfactory epithelium of G3 *mTerc^−/−^* mice. (A, B): Distribution of the mean telomere fluorescence intensity (TFI) of cells of the olfactory epithelium in 6–8 month old *mTerc^+/+^* mice (A) and of G3 *mTerc^−/−^* mice (B) (n = 4 mice per group). The dotted line shows the mean TFI of olfactory epithelial cells in mice of the two cohorts. Note that the olfactory epithelium of G3 *mTerc^−/−^* have shorter telomeres than *mTerc^+/+^* mice (P = 0.0207).

**Figure 2:**
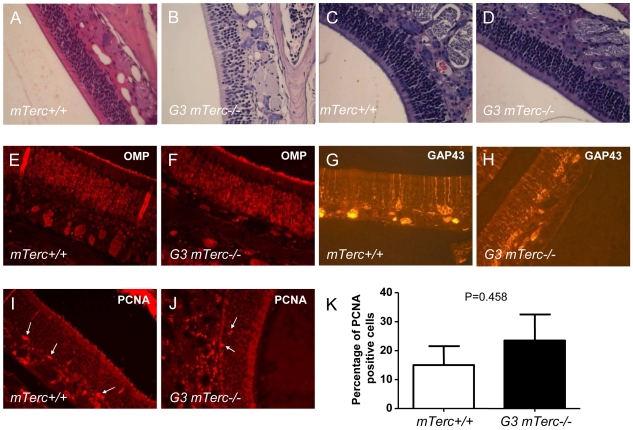
Telomere shortening does not affect homeostasis of the olfactory epithelium in aging mice. (A, B): Representative photographs of hematoxylin and eosin-stained longitudinal sections of the OE from 2–3 month old (A) *mTerc^+/+^* and (B) G3 *mTerc^−/−^* mice, and 10–12 month old (C) *mTerc^+/+^* and (D) G3 *mTerc^−/−^* mice. (E-J) Immunohistological analysis of longitudinal sections of the OE 10–12 month old (E, G, I) *mTerc^+/+^* and (F, H, J) G3 *mTerc^−/−^* mice: (E, F) Olfactory marker protein (OMP), (G, H) GAP43 and (I, J) proliferating cell nuclear antigen (PCNA). White arrows point to PCNA positive cells (G, H). (K) Histogram showing percentage of PCNA-positive cells in the OE of 10–12 month old *mTerc^+/+^* and G3 *mTerc^−/−^* mice (n = 10 mice per group, P = 0.4580).

To evaluate influences of telomere shortening on the development and postnatal maintenance of the olfactory epithelium (OE) cross section were prepared from the basal nose of 2–3 month old *mTerc^+/+^* and G3 *mTerc^−/−^* mice (n = 10 per group) and 10–12 month old *mTerc^+/+^* and G3 *mTerc^−/−^* mice (n = 10 per group). In agreement with previous studies on other organ compartments, quantitative fluorescence *in situ* hybridisation revealed significantly shorter telomeres in the OE of 6–8 month old G3 *mTerc^−/−^* compared to *mTerc^+/+^* mice (Fig. 1A, B). Histological analysis of the OE revealed a normal appearance of the OE in 2–3 month old G3 *mTerc^−/−^* mice compared to age matched *mTerc^+/+^* mice (Figure 2A,B) indicating that telomere shortening did not impair the normal development of the OE. Similarly, an analysis of cross sections from the basal nose of 10–12 month old mice did not reveal a significant influence of telomere shortening on the morphology of the OE during aging (Figure 2C, D). Moreover, immunostaining for specific differentiation markers (olfactory marker protein (OMP) for olfactory receptor neurons; growth-associated protein 43 (GAP43) for immature olfactory receptor neurons) and proliferation markers (proliferating cell nuclear antigen  =  PCNA) did not reveal a significant influence of telomere shortening on the normal differentiation and cell composition of the OE (Figure 2E-K). Together, these data indicated that telomere shortening did not impair development and postnatal maintenance of a normally differentiated OE in young and aged mice.

### Telomere shortening impairs regeneration of the olfactory epithelium in response to chemical induced damage in adult mice

**Figure 3:**
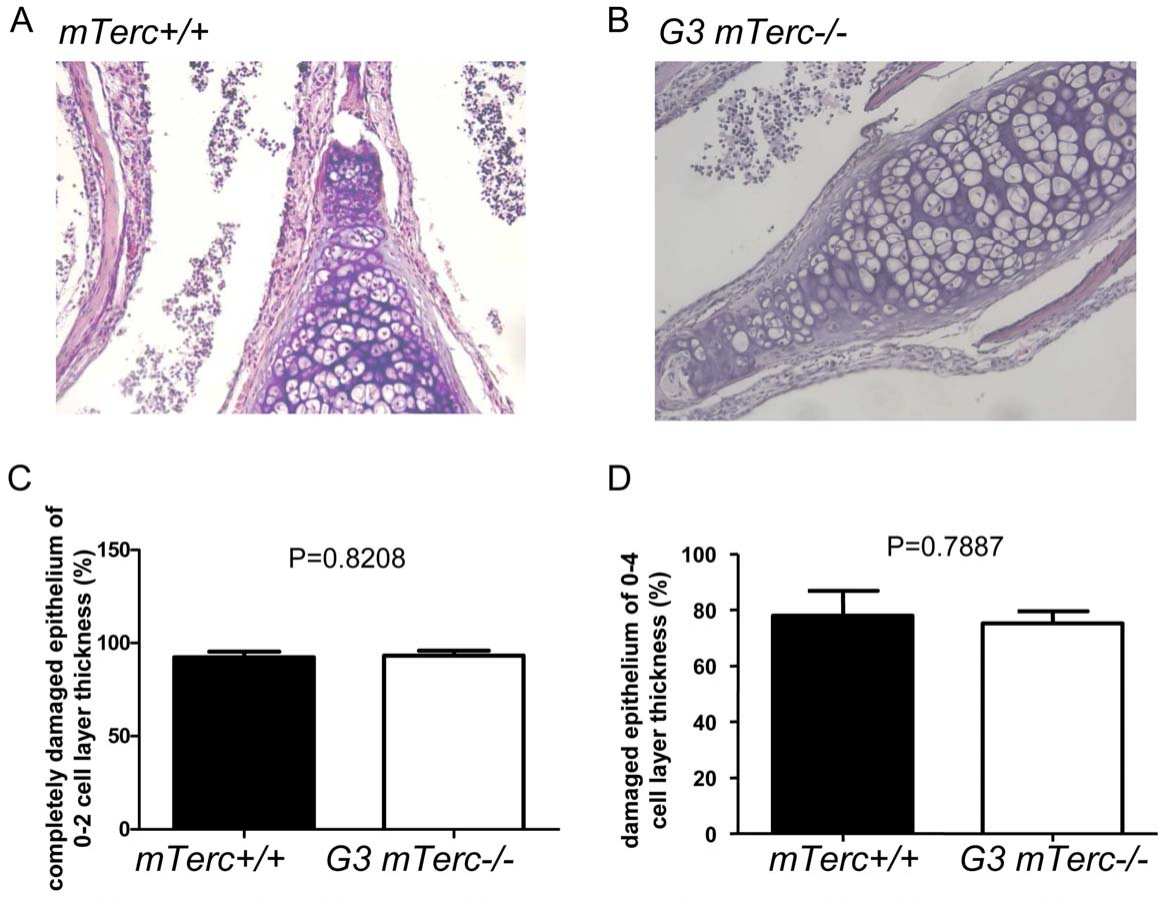
Morphological Analysis of the olfactory epithelium at day 2 after Triton-X application. (A,B) Representative photographs of hematoxylin and eosin-stained sagittal sections of the OE, two days after intranasal injection of Triton-X in 6 month old (A) G3 *mTerc^−/−^* and (B) *mTerc^+/+^* mice. There are no significant differences between the two cohorts, both showing strong damage to 80–90% of the OE. The histograms show the percentage of the chemically damaged olfactory epithelium in *mTerc^+/+^* and G3 *mTerc^−/−^* mice at two days after Triton-X induced injury: (C) percentage of damaged epithelium of 0–4 cell layer thickness (P = 0.7887), (D) percentage of completely damaged epithelium of 0–2 cell layer thickness (P = 0.8208).

To investigate the influence of telomere shortening on the regenerative response of the OE, 6 month old G3 *mTerc^−/−^* and *mTerc^+/+^* mice were treated with intranasal Triton-X (0.7%) application (n = 5 mice per group). To determine possible influences of telomere shortening on chemical induced tissue damage, cross section analysis of the OE was carried out in a cohort of mice at day 2 after Triton-X application. This analysis did not reveal significant differences between the two cohorts, both showing strong damage to 80–90% of the OE (Figure 3A-D). These data indicated that telomere shortening had no significant influence on the severity of acute OE damage in response to Triton-X application.

**Figure 4:**
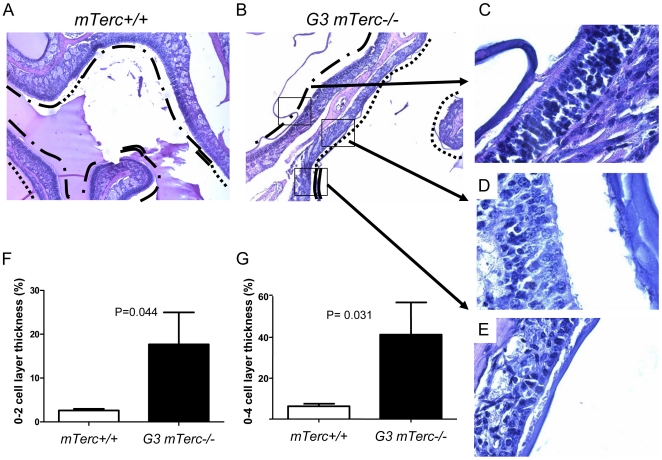
Telomere shortening impairs regeneration of the olfactory epithelium in response to injury. (A, B) Representative photographs of hematoxylin and eosin-stained sagital sections of the nasal cavity, seven days after intranasal injection of Triton-X in 6 month old (A) G3 *mTerc^−/−^* and (B-E) *mTerc^+/+^* mice. Dotted line in A and B marks incompletely regenerated epithelium of 0–2 cell layer thickness, double line marks incompletely regenerated epithelium of 3–4 cell layer thickness, dot/bar line marks completely regenerated epithelium of 5–6 cell layer thickness. Representative high-power photographs of G3 *mTerc^−/−^* mice showing (C) incompletely regenerated epithelium with 0–2 cell layer thickness (dotted line), (D) 3–4 cell layer thickness (double linier) (E) completely regenerated olfactory epithelium (E). (F, G) The histograms show the percentage of the olfactory epithelium with incomplete regeneration in *mTerc^+/+^* and G3 *mTerc^−/−^* mice at seven days after Triton-X induced injury: (F) percentage of incompletely regenerated epithelium of 0–2 cell layer thickness, (G) percentage of incompletely regenerated epithelium of 0–4 cell layer thickness.

Regeneration of the OE in response to chemical injury in mice is known to involve a strong induction of cell cycle activity leading to a near complete restoration of the OE after 7 days of injury [30]. To determine whether telomere shortening had an influence on the regenerative response after Triton-X treatment, cross sections of the OE were analyzed 7 days after Triton-X induced OE damage. In agreement with previous publications, *mTerc^+/+^* mice showed a near complete restoration of a 5–6 cell layer thick OE in large areas of the OE at this time point (Figure 4A). Regeneration of the OE was delayed in age-matched G3 *mTerc^−/−^* mice (Figure 4B-E). Specifically the OE of G3 *mTerc^−/−^* mice exhibited significantly increased areas of the OE that did not regenerate at all (0–2 cell layers, Figure 4E) or showed only incomplete regeneration (3–4 cell layer thickness, Figure 4G). These results provided the first evidence that telomere shortening impaired regenerative responses of the OE in response to chemical induced injury.

### Telomere shortening reduces the number of proliferating cells in the regenerating olfactory epithelium of adult mice in a p21-dependent manner

**Figure 5:**
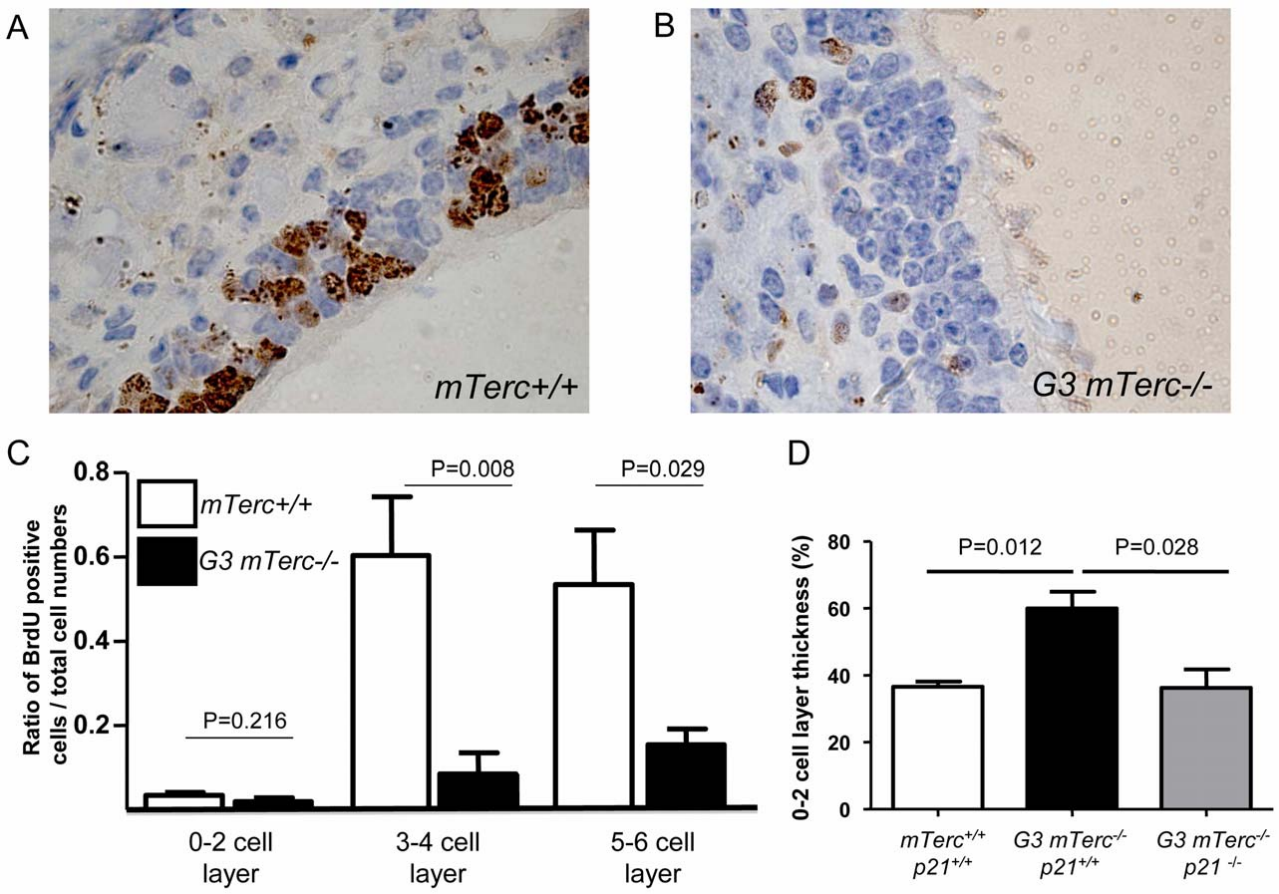
Limited proliferation potential of the OE in telomere deficient mice. (A,B) Representative photographs of BrdU-stained longitudinal sections of the olfactory epithelium, 7 days after Triton-X treatment in (A) *mTerc^+/+^* and (B) G3 *mTerc^−/−^* mice. (C) Histogram showing BrdU positive cells in the OE of G3 *mTerc^+/+^* and *mTerc^+/+^* mice. Note that there is no significant difference of the ratio of BrdU positive cells v.s. negative cells between *mTerc^+/+^* and G3 *mTerc^−/−^* mice in injured olfactory epithelium of one cell layer thickness (P = 0.216) but there was a significant reduction of BrdU positive cells in G3 *mTerc^−/−^* compared to *mTerc^+/+^* mice in injured olfactory epithelium of three cell layer thickness (P = 0.008) and 5–6 cell layer thickness (P = 0.0293), n = 5 mice per group. (D) The histogram shows the percentage of the olfactory epithelium with incomplete regeneration (0–2 cell layer thickness) in 6–8 month mice of the indicated genotypes at 7 days after Triton-X induced injury. Note that *p21* deletion rescues regenerative defects in G3 *mTerc^−/−^* mice. The cohorts in this experiment show an overall higher rate of tissue damage compared to the previous experiment depicted in Figures 3 and 4.

To analyse possible mechanisms of impaired regeneration of the OE in G3 *mTerc^−/−^* mice with short telomeres compared to *mTerc^+/+^* mice with a long telomere reserve, TUNEL (terminal deoxynucleotidyl transferase-mediated dUTP-biotin nick end labelling) staining was carried out to determine the number of apoptotic cells. In addition, mice were pulse labelled with bromodesoxyuridine (BrdU) to mark regenerating cells in S-phase of the cell cycle. Quantification of TUNEL-positive cells at day 7 after Triton-X application did not reveal significant differences in the rate of apoptosis, which was low in both cohorts (<10%, data not shown). Also the areas of impaired regeneration in the OE of G3 *mTerc^−/−^* mice did not exhibit an increase in apoptotic cells (data not shown). In contrast, BrdU staining of regenerating cells revealed a strong reduction in BrdU-positive cells in regenerating OE of G3 *mTerc^−/−^* mice compared to *mTerc^+/+^* mice, especially in areas of 2–4 and 4–6 cell thickness, at day 7 after chemical induced injury (Figure 5A-C). Together, these data indicated that telomere shortening led to impairments in OE regeneration by inhibiting the proliferative response in the regenerating areas. Staining for cell cycle inhibitors (p21) that are known to induce cell cycle arrest in response to telomere dysfunction [29], [31], [32]. Immunohistochemical staining at day 7 after Triton-X application did not reveal an increased expression of p21 in the OE of G3 *mTerc^−/−^* mice compared to *mTerc^+/+^* mice (data not shown). Similarly, quantitative PCR analysis on whole tissue lysates of the OE containing region did not show an upregulation of p21 mRNA in G3 *mTerc^−/−^* mice compared to *mTerc^+/+^* mice (data not shown). However, both techniques have limitation since preparation of the OE for IHC staining requires a 2-weeks decalcification protocol, which may damage protein epitopes. In addition, whole tissue lysates from the OE region only contains a small percentage of regenerative cells from the OE. Therefore, a genetic experiment was carried out to evaluate the functional influence of *p21* gene status (also known as *Cdkn1a*) on OE regeneration in response to chemical induced damage in 6–8 month old G3 *mTerc* ^−/−^, *p21* ^+/+^ and G3 *mTerc* ^−/−^, *p21* ^−/−^ mice. These experiments revealed that deletion of *p21* rescued the regenerative response of the OE of G3 *mTerc* ^−/−^ mice in response to chemical induced damage (Figure 5D).

## Discussion

The current study provides the first experimental evidence that telomere shortening impairs the regeneration of the olfactory epithelium in response to chemical induced injury in mice. OE proliferation was impaired in regenerating areas of the injured OE of telomere dysfunctional mice (G3 *mTerc^−/−^*) compared to *mTerc^+/+^* mice. Upregulation of the cell cycle inhibitor p21 is associated with senescence of human fibroblast in culture and also with impaired proliferation of the intestinal epithelium of G3 *mTerc^−/−^* mice (Choudhury et al. 2007, Brown et al. 1997). In this study we could not detect an upregulation of p21 in the OE of G3 *mTerc^−/−^* mice compared to *mTerc* ^+/+^ mice, which could be due to technical limitation (see above). However, our functional studies on G3 *mTerc^−^* ^/*−*^, *p21^−^* ^/*−*^ double knockout mice revealed that *p21* deletion rescued impairments in OE regeneration in response to chemical induced OE damage in telomere dysfunctional mice. Together, these experiments indicate that telomere dysfunction impairs regeneration of the OE in a *p21* dependent manner.

Previous studies have provided experimental evidence that the cellular turnover of the OE is low in wild-type mice and turnover rates decrease with aging [33]. The current study did not reveal a negative impact of telomere shortening on the maintenance of the OE under homeostatic conditions in aging G3 *mTerc^−/−^* mice. A possible explanation indicates that impairments in cell proliferation can be compensated in organ systems with low rates of cell turnover. In agreement with this assumption, telomere dysfunction induced atrophy of tissues in G3 *mTerc^−/−^* mice predominantly affects organ systems with high rates of cell turnover [28], [34]. The data on impaired regeneration of the OE in chemically injured G3 *mTerc^−/−^*mice indicate that compensatory regeneration may exhaust in the context of increased rates of proliferation that are required to regenerate the tissue in response to injury. However, we also recognized that the regeneration of the OE of G3 *mTerc^−/−^* mice could be completed at later time points after chemical injury (3 weeks, data not shown) indicating that an acute, single injury can be restored despite impairments in regeneration. However, this healing occurs only with a significant delay. It remains to be investigated whether telomere shortening and p21 upregulation may contribute to the evolution of OE dysfunction in elderly humans. According to the current study this mechanisms could especially contribute to the evolution OE atrophy in response to acute or chronic damage, such as in response to surgery or infections.

## Materials and Methods

### Animals

*mTerc^−/−^* [27], *p21^−/−^* [35] and as control *mTerc^+/+^* mice on C57BL/6J background were used in this study. *mTerc^+/−^* mice were crossed with *p21^−/−^* mice. Heterozygous offsprings were crossed with each other to generate G1 *mTerc^−/−^ p21^−/−^* mice. Those mice where crossed until the third generation of the Telomerase knockout G3 *mTerc^−/−^ p21^−/−^*. Mice were kept in a pathogen free environment where fed with ad libidum access to food and water. The animal experiments were approved by the government of the state of Baden-Württemberg (animal protocol number 35/915.81–3–919).

### Lesions

Mice from G3 *mTerc^−/−^* mice and *mTerc^+/+^* mice were treated with a single intranasal irrigation with 0.7% of Triton-X in 0.15% NaCl. Control mice were treated with same volume of 0.9% saline.

### *In vivo* BrdU incorporation assay

*In vivo* labelling of proliferating cells was performed by BrdU labelling [i.p. injection of BrdU 30 mg/kg body weight]. G3 *mTerc^−/−^* mice and *mTerc^+/+^* mice were injected with a bromodeoxyuridine (5-bromo-2-deoxyuridine; BrdU) solution every day and 7 days later they were killed and the head without skin were collected and fixed with 4% paraformaldehyde overnight.

### Decalcification

The facial bones were decalcifyed with 15% EDTA 0.5% PFA in PBS (pH 8.0). Decalcification is carried out at 4°C with gentle agitation. The fluid is renewed every 1–2 days until the calcium salts were completely removed.

### Telomere length measurement by quantitative fluorescence *in situ* hybridization

Paraffin slides were unmasked and than incubated in Pepsine solution for 10 min at 37°C (100 mg Pepsine; 84 µl HCl 37% up to 100 ml H_2_O) and washed in PBS. The hybridisation mix (10 mM Tris pH 7,2; MgCl_2_ buffer : 7,02 mM Na_2_HPO_3_, 2,14 mM MgCl_2_, 0,77 mM citric acid; 70% deionized formamide; 0,5 µg/ml PNA probe 5̀-Cy3-CCC TAA CCC TAA CCC TAA-3′ Panagene; 0,25% Roche blocking reagent) was added to the sections and denatured at 80°C for 3 min followed by 2 h incubation in the dark. Slides were incubated in 70% formamide, 10 mM Tris (pH 7,2), 0,1% BSA two times for 20 min and washed 3 times in TBS-Tween (0,2%). Relative telomere length was measured by the TFL analysis software program [36].

### Histology and Immunohistochemical stainings

Immunofluorescence was performed on 5 µm thick paraffin sections. Sections were deparaffinized and rehydrated in series of ethanol and unmasked in 1 mM sodium citrate buffer by heating at boiling temperature for 5 min and then heating at sub-boiling temperature for 10 min and then allowed to cool down at RT for 40 min. The slides were then washed in PBS twice and incubated with primary antibody for OMP (Biosensis, 1∶300 dilution), GAP 43 (Biocompare, 1: 300 dilution), BrdU (BectonDickinson 1∶100 dilution) and PCNA (Calbiochem, 1∶50 dilution) either over night at 4°C or for 2 h in humid chamber at room temperature. The slides were washed twice with PBS before treated with secondary antibody, anti-mouse IgG Cy3-conjugated (Cat No. C2181 Sigma-Aldrich, 1∶300 dilution). Bound antibodies were visualized using 1 mg/ml 3,3′ diaminobenzidine tetrahydrochloride (Sigma Chemical) for BrdU staining. Sections were dehydrated through a series of graded alcohols and Microclearing and mounted with Vitro-Clud (Microm Microtech).

The evaluation was made as the percent of positive cells and was counted in 10 low power fields (200 x) per mouse.
